# The paradox of hnRNPK: both absence and excess impair skeletal muscle function in mice

**DOI:** 10.1186/s13395-025-00393-3

**Published:** 2025-08-07

**Authors:** Yongjie Xu, Yuxi Wang, Xiaofang Cheng, Mengjia Zhang, Nuo Chen, Jiahua Guo, Yueru Huang, Quanxi Li, Tianyu Li, Tiantian Meng, Cencen Li, Pengpeng Zhang, Haixia Xu

**Affiliations:** 1 College of Life Science, Xinyang Normal University, Xinyang, 464000 China; 2 Institute for Conservation and Utilization of Agro-Bioresources in Dabie Mountain, Xinyang Normal University, Xinyang, 464000 China

**Keywords:** HnRNPK, Skeletal muscle, Knockout, Overexpression, Mice, Muscle atrophy

## Abstract

**Background:**

The RNA-binding protein hnRNPK is essential for animal growth and development, with a particular emphasis in myogenesis. Despite its importance, the precise mechanisms by which hnRNPK influences skeletal muscle physiology and development remain inadequately characterized.

**Methods:**

To explore its regulatory function, we developed a *Myf5*-cre-mediated myoblast precursor-specific knockout mouse model (*Hnrnpk* mKO), an *Acta1*-CreEsr1-mediated myofiber-specific inducible knockout mouse model (*Hnrnpk* aKO), and an AAV9-mediated skeletal muscle-specific overexpression mouse model (AAV9-hnRNPK). Morphological alterations in skeletal muscle were assessed using hematoxylin and eosin (HE) staining subsequent to hnRNPK knockout or overexpression. Global gene expression changes in the tibialis anterior (TA) muscle were assessed via RNA sequencing (RNA-seq). Furthermore, reverse transcription quantitative polymerase chain reaction (RT-qPCR), western blot analysis, immunofluorescence, immunohistochemistry, co-immunoprecipitation (Co-IP), dual luciferase analysis, and reactive oxygen species (ROS) detection were utilized to elucidate the molecular mechanisms by which hnRNPK contributes to skeletal muscle development.

**Results:**

Our findings indicate that the ablation of hnRNPK in myoblast precursors significantly impairs muscle development, disrupts fetal myogenesis, and results in embryonic lethality. In adult mice, both the loss and gain of hnRNPK function led to reduced muscle mass, decreased fiber size, and compromised skeletal muscle homeostasis. Importantly, the knockout of hnRNPK had a more substantial impact on skeletal muscle development compared to its overexpression, with myofiber-specific knockout leading to mortality within two weeks. Mechanistically, hnRNPK deficiency was associated with increased apoptosis and muscle atrophy, characterized by elevated expression of genes involved in apoptosis, muscle atrophy, and protein catabolism, along with impaired muscle contraction and extracellular matrix (ECM) organization. Conversely, hnRNPK overexpression was correlated with enhanced ferroptosis pathway and improved ECM organization, but was also associated with reduced oxidative phosphorylation and protein synthesis. The overexpression likely promotes ferroptosis via the hnRNPK/P53/Slc7a11/Gpx4 pathway, thereby accelerating muscle aging and reducing muscle mass.

**Conclusion:**

In conclusion, our findings underscore the critical importance of precise hnRNPK expression levels in maintaining skeletal muscle health. Both deficiency and overexpression of hnRNPK disrupt skeletal muscle development, highlighting its pivotal role in muscle physiology.

**Clinical trial number:**

Not applicable.

**Supplementary Information:**

The online version contains supplementary material available at 10.1186/s13395-025-00393-3.

## Background

Heterogeneous ribonuclear proteins (hnRNPs) are a family of evolutionarily conserved RNA-binding proteins that play pivotal roles in gene transcription, RNA splicing, mRNA stability, and protein translation [1, 2]. Within this family, hnRNPK is distinguished as one of the most extensively studied members, recognized for its essential role in nucleic acid metabolism and its involvement in pathologies such as neurodegenerative disorders and cancers. hnRNPK is uniquely defined by the presence of three KH domains, a nuclear localization signal (NLS), and a KNS domain, which collectively facilitate its shuttling between the nucleus and cytoplasm. This dynamic localization enables hnRNPK to engage in a diverse range of cellular processes, including chromosome remodeling, gene regulation, and signal transduction, all of which are essential for normal growth and development [3, 4].

The significance of hnRNPK in developmental processes is highlighted by the observation that its absence results in embryonic lethality prior to day 13.5 in mice. In humans, mutations in HNRNPK are linked to Au-Kline Syndrome (AKS), a disorder characterized by growth delays, intellectual disability, and muscle weakness [5]. These findings underscore the pivotal role of hnRNPK in the maintenance of cellular and organismal homeostasis. Notably, hnRNPK is ubiquitously expressed across various mammalian tissues, including skeletal muscle, where it has been implicated in muscle development and regeneration [6]. Specifically, hnRNPK is indispensable for myogenic differentiation, a process essential for muscle repair and maintenance, through its interaction with MyoD, a key transcription factor that activates the expression of muscle-specific genes [7]. Furthermore, hnRNPK plays a critical role in regulating RNA splicing and stability, processes that are crucial for the appropriate expression of myogenic factors and the formation of muscle fibers [8]. Despite these advancements, the precise mechanisms by which hnRNPK influences skeletal muscle physiology and development remain not fully elucidated. Previous studies, including our investigations utilizing C2C12 cells and CRISPR/Cas9 technology, have demonstrated that hnRNPK is essential for myoblast proliferation and differentiation [9]. Additionally, our research has revealed that hnRNPK stabilizes *Cdkn1a* mRNA, a vital regulator of muscle progenitor cell proliferation and differentiation [10]. However, the broader implications of hnRNPK in muscle homeostasis, particularly in the context of muscle mass maintenance and aging, warrant further investigation.

This study seeks to reveal the potential roles and mechanisms of hnRNPK in skeletal muscle function through the utilization of Myf5-cre and Acta1-CreEsr1-mediated Hnrnpk deletion mouse models, in conjunction with an adeno-associated virus serotype 9 (AAV9) overexpression system. Our findings demonstrate that hnRNPK is crucial for myogenesis and the preservation of muscle mass and homeostasis. Both the deficiency and overexpression of hnRNPK in mice result in the activation of the P53 pathway, leading to disrupted muscle homeostasis and a reduction in muscle mass. Specifically, hnRNPK deficiency induces muscle cell death and aberrant expression of atrophy-related genes such as MURF1 and MAFbx, culminating in muscle atrophy. In contrast, hnRNPK overexpression inhibits the SLC7A11/GPX4 pathway, thereby activating the ferroptosis pathway and promoting muscle aging, which also leads to a decrease in muscle mass. These findings not only enhance our understanding of hnRNPK’s role in muscle health but also propose potential therapeutic targets for chronic muscular disorders. By elucidating the dual roles of hnRNPK in muscle atrophy and aging, this research lays the groundwork for novel interventions aimed at preserving muscle function and mitigating age-related muscle decline.

## Materials and methods

### Animal used

All animal experiments in this study were approved by the Institutional Review Board of Xinyang Normal University. Mice were hosted on a 12 h light/12 h dark cycleat 50 ~ 70% humidity and a temperature of 22 ± 2 ℃, with unlimited access to food and water. *Hnrnpk* ^*loxP/loxP*^ mice were previously described [11]. To create mice with *Hnrnpk* knockout in skeletal muscle lineage cells (*Hnrnpk* mKO), *Hnrnpk* ^*loxP/loxP*^ mice were crossed with *Myf5*-*Cre* mice (The Jackson Laboratory, stock no.007893), where Cre is expressed in brown fat and skeletal precursor cells under the Myf5 promoter. Myofiber-specific *Hnrnpk* knockout mice were created by crossing inducible mouse model *Acta1*-CreEsr1 mice (The Jackson Laboratory, stock no. 031934) with *Hnrnpk* ^loxP/loxP^ mice, resulting in *Acta1*-CreEsr1; *Hnrnpk* ^loxP/loxP^ mice (Fig. S1), designated as conditional homozygous knockout (*Hnrnpk* aKO) mice. Their *Hnrnpk* ^flox/flox^ littermates served as controls (WT). Mice were genotyped by PCR using standard protocols.

### Tamoxifen injection and skeletal muscle injury

Once the proper genotype was obtained, 6-week-old male mice were administered intraperitoneally at daily injection solution dosage of 1 mg/20 g of Tamoxifen (TMX, Sigma) for five consecutive days. Muscles were then harvested at 14 days post injection, and stored at -80 °C prior to sectioning.

### AAV9 virus packaging and local injection into muscles

We generated pHBAAV-CMV-hnRNK-T2A-mcherry plasmid (AAV9-hnRNK), in which the CMV promoter is used to drive the expression of hnRNK, and the mouse *Hnrnpk* cDNA (GenBank accession no. NM_001301341.1) was obtained from the total RNA from the skeletal muscle. pHBAAV-CMV-T2A-mcherry was used as control (AAV9-NC). AAV9 virus was packaged by Hanbio Techonology Company (Shanghai, China). For local intramuscular(TA muscle)AAV9 delivery, 6 Weeks old male mice (*n* = 6) were randomized into AAV9- hnRNK group (TA of right leg) or AAV9-NC group (TA of left leg) and anesthetized deeply with isoflurane. The mice were injected intramuscular to bilateral TA muscle at dose of 5*10^10^ vector genome (vg) in a final volume of 50 µL per set (10^11^ vg/group). The mice were sacrificed and analyzed after 4-week intervention.

### RT-qPCR

Total RNA was extracted from skeletal muscle using Trizol reagent (Takara, Dalian, Japan) and a reverse transcription (RT) reaction was performed using M-MLV Reverse Transcriptase (Invitrogen, Waltham, MA, USA) according to the manufacturers’ instructions. RT-qPCR was carried out on the Bio-Rad C1000 Touch PCR System. Gene-specific primers (detailed in Table S1) were used to determine the relative expression levels of the candidate target genes according to the standard-curve methodology, which were quantified relative to the expression of the mice *Gapdh* gene and and *18 S rRNA*, by employing an optimized comparative Ct value method (ΔΔCt), and the expression level was calculated as 2^^(−ΔΔCt)^ to compare the relative expression. Each assay was performed in triplicate with 3 independent replicates.

### Histological examination and immunofluorescence

Muscle tissues were collected and weighed, fixed in 4% PFA (paraformaldehyde) solution, and then embedded in paraffin wax to cut into 5-µm-thick tissue slices using a rotary microtome. Six slices of each skeletal muscle sample were stained with H&E (hematoxylin-eosin) for histological analysis. Cross sections were deparaffinized with environmentally friendly dewaxing transparent liquid and rehydrated in a descending ethanol series. Then Immunofluorescence and immunohistochemistry staining were performed according to our previous protocol [11]. The following primary antibodies information was show in Table S2. The images were visualized using a Nikon 80i microscope with NISElements software (Nikon, Tokyo, Japan).

### Western blotting and immunoprecipitation

The skeletal muscle protein samples were prepared using RIPA lysis buffer (Beyotime, China) with the cocktail of protease inhibitor (Beyotime, China). Protein was quantified using Enhanced BCA Protein Assay Kit (Beyotime, China). Then, protein samples were denatured by heating at 100 °C, separated on 12.5% SDS-PAGE gels and transferred to PVDF membranes. The blotted membranes were blocked with 5% non-fat dry milk for 1 h at room temperature, and then incubated with appropriate primary antibodies (Table S2) overnight at 4 °C. Finally, the membranes incubated with HRP-labeled Goat Anti-Mouse IgG or HRP-labeled Goat Anti-Rabbit IgG (Beyotime, China) as secondary antibodies at a dilution of 1:1000 and detected by the enhanced chemiluminescence (ECL) System (Beyotime, China). Images were acquired and quantified using a FluorChem M imaging system (ProteinSimple, USA). For immunoprecipitation, cells were washed with PBS and lysed in ice-cold IP lysis buffer. Protein incubated 1000 µg of musle lysate with the hnRNPK or HA antibody-conjugated beads at 4 °C for 4 h. The immune-complexes were washed 3 times with IP lysis buffer before being resolved by SDS-PAGE and immunoblotted with the indicated antibodies.

### Plasmid construction, cell lines and cell culture

The full length *p53* gene was cloned into the pKH3 vector with PstI and SalI sites. The *Slc7a11* promoter was cloned into the pGL3 vector with SmaI and HindIII sites. The full length *Hnrnpk* gene was cloned into the pcDNA3.1(+) vector with XhoI and NotI sites. The *p53* gene was amplified with Seamless Cloning Kit (D7010FT, Beyotime, China) using the following primers: *p53* sense primer (5′-ctcggttctaagcttctgcagATGACTGCCATGGAGGAGTCAC-3′), *p53* anti-sense primer (5′-tgggtacattctagagtcgacGTCTGAGTCAGGCCCCACTTT-3′). The *Slc7a11* promoter was amplified with Seamless Cloning Kit (D7010FT, Beyotime, China) using the following primers: *Slc7a11* sense primer (5′-tcttacgcgtgctagcccgggACCCTTACTTTTCAGCACTTTTCC-3′), *Slc7a11* anti-sense primer (5′-cagtaccggaatgccaagcttTAAAATGCATCTCTGAGGAAAGGA-3′). The *Hnrnpk* gene was amplified using the following primers: *Hnrnpk* sense primer (5′-CCGCTCGAGATGGAAACGGAACAACCAG-3′), *Hnrnpk* anti-sense primer (5′- TCCCCGCGGGAATCCTTCAACATCTGCA-3′). The C2C12 and HEK-293T cell lines were cultured in Dulbecco’s modified Eagle’s medium (DMEM) with 10% fetal bovine serum (FBS) supplemented with 100 U/ml penicillin and 100 mg/ml streptomycin. Cells were transfected using the Lipofectamine 3000 reagent (Life Technologies) according to the manufacturer’s instructions. All cells were maintained under standard culture conditions (37 °C, 5% CO_2_).

### Co-immunoprecipitation (Co-IP) assays

HEK-293T cells transiently transfected with pKH3-HA-P53 were seeded in 100 mm dishes. The cells were lysed in RIPA Lysis Buffer (P0013D, Beyotime, China) supplemented with protease inhibitor cocktail (P1010, Beyotime, China). Lysates were incubated with primary antibody overnight at 4 °C. Then, 40 µl of a 1:1 slurry of protein A/G Agarose (P2012, Beyotime, China) and a specific antibody (HA or hnRNPK) were added to the cells for ≥ 4 h at 4 °C. The immunoprecipitates were washed four times with lysis buffer and boiled with sample loading buffer and then analyzed by western blotting.

### Reactive oxygen species (ROS) detection

Intracellular levels of ROS in C2C12 myocytes were measured using Reactive Oxygen Species Assay Kit (S0033S, Beyotime, China) according to the manufacturers’ instructions. In brief,

After C2C12 myocytes transfected with pcDNA3.1 or pcDNA3.1-Hnrnpk r for 48 h, the cells in 48-well plates were incubated with 10 µM DCFH-DA fluorescence probe in Krebs Ringer buffer solution for 20 min at 37 ℃. After washing with PBS 3 times, the intensity of intracellular ROS was monitored using a Nikon 80i microscope with NISElements software (Nikon, Tokyo, Japan) at excitation and emission wavelengths of 488 and 525 nm, respectively.

### Double luciferase analysis

All of the transfections were done in triplicate in 24-well plates. Approximately, 1 × 10^3^ cells/well was seeded 24 h before transfection. The pcDNA3.1 or pcDNA3.1-*Hnrnpk* and pGL3-*Slc7a11* promoter plasmids were co-transfected into HEK-293T cells using Lipofectamine 3000 (Life Technologies, Inc.). Luciferase assays were performed using the Dual Luciferase Assay System (Beotime, Rg027, China) that already contains an internal control detectable simultaneously with the luciferase reporter gene.

### RNA-Seq library preparation and analysis

RNA was isolated from TA muscle using RNAiso Plus reagent (TaKaRa, Japan). The qualified total RNA was further cleaned up by using RNAClean XP Kit (Beckman, Krefeld, Germany) and RNase-Free DNase Set (QIAGEN, Hilden, Germany). After passing through the quality inspection, the RNA-Seq library was constructed using the VAHTS Stranded mRNA-seq Library Prep Kit for Illumina^®^ (Vazyme, NR602-02), and sequenced on the Illumina Hiseq 2000/2500 NextSeq with paired-end sequencing in Shanghai Bohao Biotechnology Co., Ltd. After prefiltering the raw data by removing the joint sequence and low-quality reads, the preprocessed reads were conducted to align the mouse genome GRCm38.p4 (mm10) using Hisat2 estimated by (version: 2.0.4) alignment software for genome mapping. The expression level of each gene was normalized by FPKM using Stringtie software (version 1.3.0) [12]. Genes exhibiting a *|*Fold Change*|* ≥ 1.5 and *q* ≤ 0.05 were considered to be significantly differentially expressed in the testes derived from knockout or overexpression group compared to in control groups. The biological functions of identified differentially expressed genes were annotated using Metascape bioinformatics analysis website tools (https://metascape.org) [13]. Gene Set Enrichment Analysis (GSEA) was visualized using the OmicShare tools, a free online platform for data analysis (http://www.omicshare.com/tools).

### Statistical analysis

All the experiments were carried out at least in triplicate and the values were shown as mean ± SEM. Statistical significances were measured based on *t-test* (two groups) or one-way ANOVA using the SPSS17.0 software. *p* ≤ 0.05 (*) was considered as significant difference, and *p* ≤ 0.01 (**) was considered as a very significant difference between the two groups.

## Results

### Myf5-Cre mediated hnrnpk conditional knockouts results in perinatal lethality

**Fig. 1:**
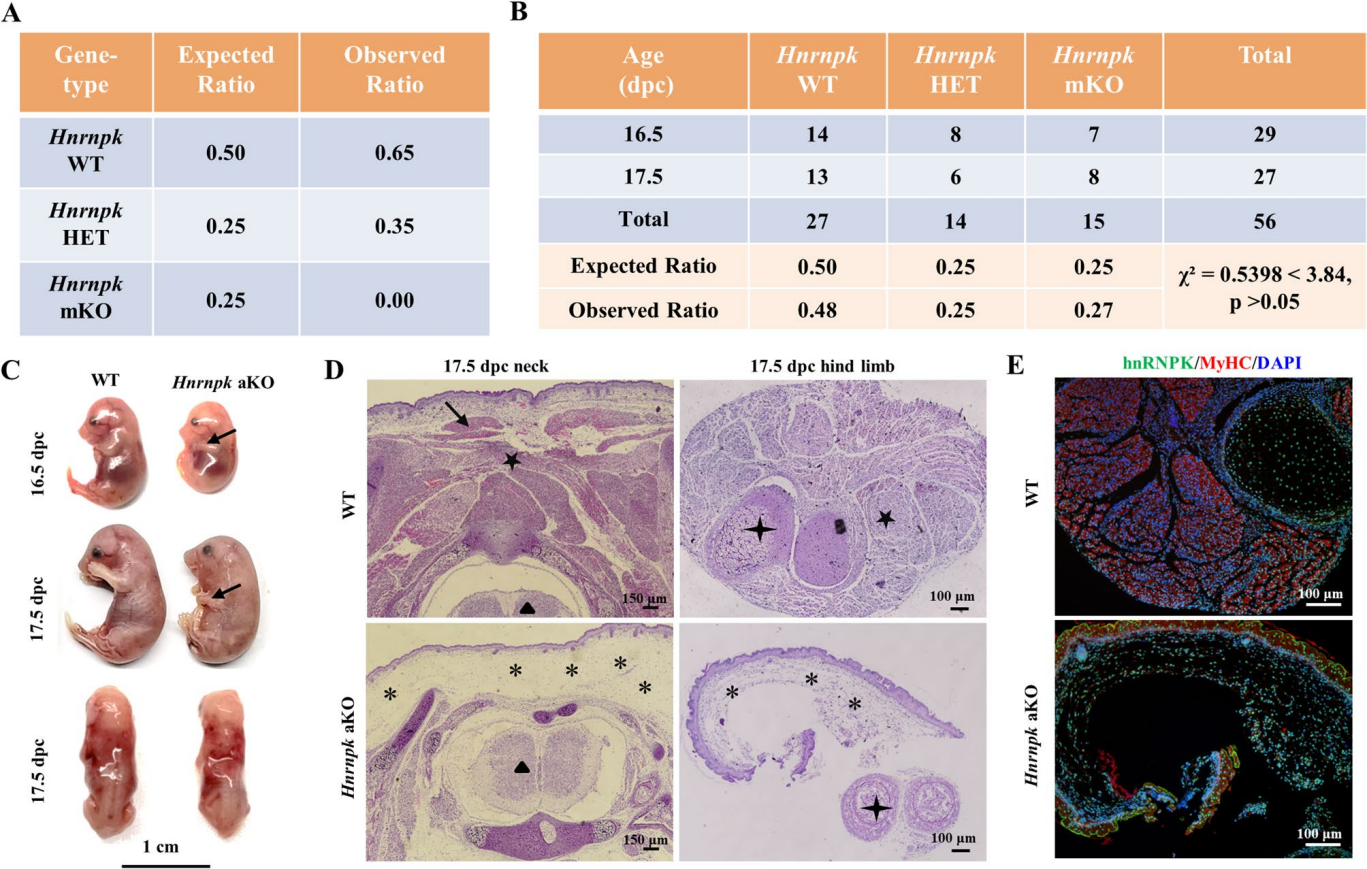
Myf5-Cre-Mediated *Hnrnpk* conditional knockouts results in perinatal lethality. (**A**) Skeletal muscle-specific *Myf5-Cre/Hnrnpk* conditional knockout (*Hnrnpk* mKO) is lethal prior to weaning. The proportion of various genotypes obtained an N of 175 by mating between *Hnrnpk* ^*LoxP/−*^; *Myf5-Cre* and *Hnrnpk*^*LoxP/LoxP*^. We expect that 25% of pups to be homozygous for *Hnrnpk*-deficient alleles, 25% of pups should be heterozygous (HET), and 50% should be wild type (WT). Litters were genotyped at weaning and the observed numbers were recorded. There was no significant reduction in litter size between birth and weaning. (**B**) Skeletal muscle-specific *Hnrnpk* conditional knockout does not alter Mendelian ratios prior to birth. (**C**) Images of a *Hnrnpk* homozygous mutant mouse. *Hnrnpk* mKO mice displayed a smaller body size, slightly abnormal hunched posture and dropping forelimbs (black arrow). (**D**) The cervical region and leg transverse sections of E17.5 WT and *Hnrnpk* mKO embryos stained with H&E, revealing reduction of myocytes and general affliction of some muscles. Marked subcutaneous edema is most visible in the cervical region and leg (star). The dorsal muscles and thigh muscle (black pentagram) are significantly diminished in the *Hnrnpk* mKO. There is a lack of brown adipose tissue in *Hnrnpk* mKO (black arrow). Black triangles and asterisks represent vertebrae and leg bones, respectively. Bar = 100 μm. (**E**) Detection of hnRNPK (green) by immunofluorescence (IF) staining in transversal hind limb sections of *Hnrnpk* mKO and control fetuses at E17.5. Muscle fibers were visualized with myosin heavy chain (MyHC) antibody (red), nuclei were stained with DAPI (blue), bar = 100 μm

To explore hnRNPK’s role in skeletal muscle development, we crossed *Hnrnpk* ^*LoxP/LoxP*^ mice with *Myf5*-Cre mice to conditionally delete *Hnrnpk* in muscle progenitor cells (Fig. S1). Surprisingly, genotyping of 175 weaned pups from 30 litters at 3 weeks revealed no homozygous conditional knockout mice (*Hnrnpk* mKO) (Fig. 1A). While some pups died shortly after birth, likely due to respiratory issues, no significant mortality occurred between postnatal day 1 and weaning. At E16.5 to E17.5, *Hnrnpk* mKO embryos appeared at expected Mendelian ratios (Fig. 1B) but exhibited reduced size, a hunched posture, and drooping forelimbs, indicative of severe muscle loss and neuromuscular defects (Fig. 1C). H&E staining at E17.5 confirmed significant skeletal muscle disruption and subcutaneous edema in *Hnrnpk* mKO embryos (Fig. 1D). Immunostaining revealed the absence of hnRNPK protein in MyHC-positive myofibers of *Hnrnpk* mKO fetuses, while hnRNPK expression persisted in non-myogenic cells such as fibroblasts and osteoblasts (Fig. 1E and Fig. S2). These results demonstrate that hnRNPK is essential for skeletal muscle development, and its loss in skeletal muscle leads to early postnatal lethality in most mice.

### The absence of hnRNPK leads to progressive muscle fiber atrophy and loss of muscle mass

**Fig. 2:**
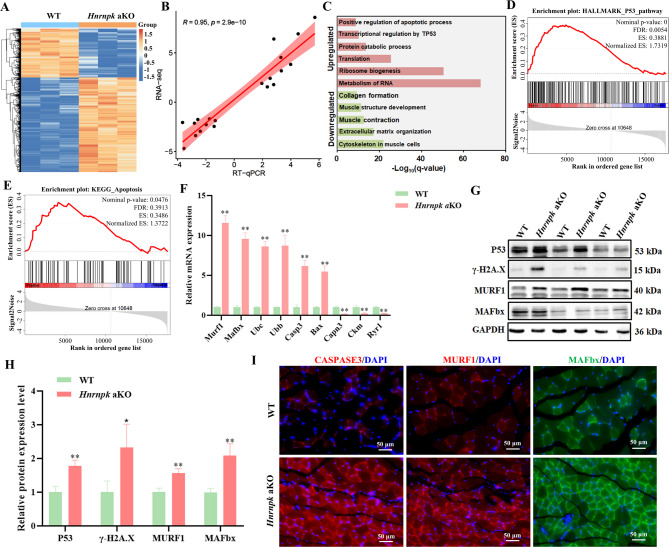
The absence of hnRNPK leads to progressive muscle fiber atrophy and loss of muscle mass. (**A**) Representative photographs show the development of kyphosis in *Hnrnpk* aKO mice. (**B**) Induced knockout of hnRNPK in muscle fibers resulted in significant weight loss in mice (*n* = 6). (**C**) Skeletal muscles wasting in the *Hnrnpk* aKO mice can be seen in whole body muscles. (**D**) Muscle mass of TA, EDL, SOL and GAS muscles were comparative between WT and *Hnrnpk* aKO mice (*n* = 6). (**E**) Comparison of representative samples of dissected TA muscle between WT and *Hnrnpk* aKO mice. (**F**) Immunoblotting detecting the expression of protein hnRNPK in WT control and *Hnrnpk* aKO TA muscle. The expression level of GAPDH was used as an internal control. (**G**) hnRNPK was significantly downregulated in the *Hnrnpk* aKO mice after inducing knockout for 4 weeks (*n* = 3). (**H**) Representative TA muscles stained with hematoxylin and eosin (H&E) from WT control and *Hnrnpk* aKO group. (**I**) Quantification of muscle fibre SCA in TA (*n* = 6). (**J**) Frequency of distribution for muscle diameter (µm) of TA muscle (*n* = 6)

To confirm hnRNPK’s in vivo role, we generated inducible myofiber-specific *Hnrnpk* knockout mice (*Hnrnpk* aKO) by crossing *Acta1*-CreEsr1 mice with floxed *Hnrnpk* mice. *Hnrnpk* deletion was induced in 8-week-old males via tamoxifen (TMX) injection (100 mg/kg i.p. for 5 days). Unexpectedly, *Hnrnpk* aKO mice began dying 15–18 days post-knockout, exhibiting kyphosis (Fig. 2A) and significant weight loss due to muscle weakness (*p* < 0.05) (Fig. 2B). These findings indicate that hnRNPK is crucial for muscle maintenance and development. Fourteen days after *Hnrnpk* ablation, the muscles of knockout mice appeared dark red compared to those of the WT group (Fig. 2C). The weights of the tibialis anterior (TA), gastrocnemius (GAS), and extensor digitorum longus (EDL) muscles were significantly reduced in the knockout group compared to the WT group (*p* < 0.05), while the soleus (SOL) muscle weight remained unchanged (*p* > 0.05) (Fig. 2D and **E**). Western blotting confirmed a 70% reduction in hnRNPK protein in TA tissue of the knockout group, validating the TMX-induced knockout (Fig. 2F and **G**). H&E staining revealed notable morphological differences and a 30% decrease in muscle cross-sectional area in knockout mice (Fig. 2H and **I**), suggesting that hnRNPK knockout impairs muscle development. Furthermore, the number of muscle fibers with diameters below 40 μm significantly increased (*p* < 0.05), with diameters concentrated between 30 and 40 μm, unlike the control group, where diameters were predominantly 40–70 μm (Fig. 2J).

**Fig. 3:**
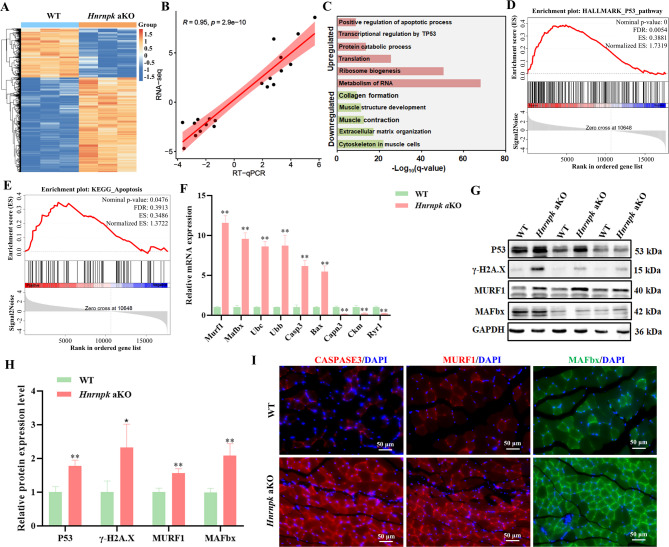
RNA-Seq analysis of the effects of hnRNPK knockout on skeletal muscle development in mice. (**A**) Heatmap of the expression values (normalized as transcripts per million) of the DEGs in the WT and *Hnrnpk* aKO groups TA sample using RNA-Seq. (**B**) Correlation between the fold-change in RNA-Seq analysis and those obtained by RT-qPCR analysis for the 18 selected genes in TA muscles. (**C**) Bar charts showing the enriched GO terms for upregulated and downregulated DEGs between WT and *Hnrnpk* aKO groups in TA muscles. **(D**) GSEA of P53 pathway genes in TA muscle upon *Hnrnpk* knockout. **(E**) GSEA of apoptosis genes in TA muscle upon *Hnrnpk* knockout. (**F**) The transcriptional levels of muscle atrophy, protein catabolic process, and apoptosis related genes were detected by RT-qPCR in TA muscles from WT control and *Hnrnpk* aKO group (*n* = 3). (**G**) Western blot analysis of P53, γH2A.X, ASZ1, MURF1 and MAFbx expression in TA muscles from WT control and *Hnrnpk* aKO group. (**H**) Statistical analysis of the Western blot results (*n* = 3). All bar and line chart results are expressed as mean values ± SEM, and a paired two-tailed Student’s t-test was used to statistical analysis. ∗∗*p* < 0.01, ∗*p* < 0.05; NS, not significant. (**I**) Immunofluorescence detection of CASPASE3 (red), MURF1 (red) and MAFbx (green) expression in TA muscles from WT control and *Hnrnpk* aKO group

To explore the molecular mechanisms underlying these phenotypes, RNA-Seq was performed on TA muscles from knockout mice 14 days post-knockout. This analysis identified approximately 5,707 differentially expressed genes (DEGs) (Fig. 3A and Table S3). Among these, 2,869 genes were up-regulated and 2,838 were down-regulated. Analysis of 18 muscle fiber-related genes showed a strong correlation between RT-qPCR and RNA-Seq data (Pearson *R* = 0.95, *p* = 2.9e-10) (Fig. 3B, Fig. S3A and B), validating the RNA-Seq findings. To identify the biological pathways affected by hnRNPK depletion, we performed functional enrichment analysis using Metascape. Up-regulated DEGs were associated with RNA metabolism, ribosome biogenesis, translation, protein catabolism, TP53-mediated transcription, and apoptosis regulation. Down-regulated DEGs were primarily involved in cytoskeleton assembly, extracellular matrix organization, muscle contraction, muscle development, and collagen formation (Fig. 3C and Table S4). These results suggest that hnRNPK influences muscle development by modulating downstream genes and pathways. Gene Set Enrichment Analysis (GSEA) further revealed significant enrichment of altered transcripts in the P53 pathway (Fig. 3D), apoptosis (Fig. 3E), proteasome complex, muscle contraction, and extracellular matrix organization (Fig. S4A). P53 is a transcription factor that regulates genes controlling apoptosis, senescence, and cell growth, all of which are critical for maintaining cellular homeostasis [14, 15]. hnRNPK co-activates P53 to maintain cellular stability, and disruptions in their interaction can lead to cellular dysfunction [16]. Reduced hnRNPK levels may induce apoptosis, disrupt muscle homeostasis, and impair muscle function through the P53 pathway.

RT-qPCR analysis confirmed increased expression of genes associated with muscle atrophy (*Murf1* and *Mafbx*), protein catabolism (*Ubc* and *Ubb*), and apoptosis (*Casp3* and *Bax*), alongside decreased expression of genes related to muscle structure and contraction (*Capn3*, *Ckm*, and *Ryr1*) in *Hnrnpk* knockout mice (Fig. 3F). Protein levels of P53, γ-H2A.X, MURF1, and MAFbx were significantly lower in *Hnrnpk* aKO mice compared to WT mice (Fig. 3G and **H**). Immunostaining further revealed low levels of CASPASE3, MURF1, and MAFbx proteins in WT muscle fibers but elevated levels in *Hnrnpk* knockout skeletal muscle (Fig. 3I). Collectively, these findings underscore hnRNPK’s essential role in skeletal muscle development and homeostasis, with its absence leading to disrupted muscle function and development.

### AAV9 mediated overexpression of hnRNPK in skeletal muscle accelerants muscle aging

**Fig. 4:**
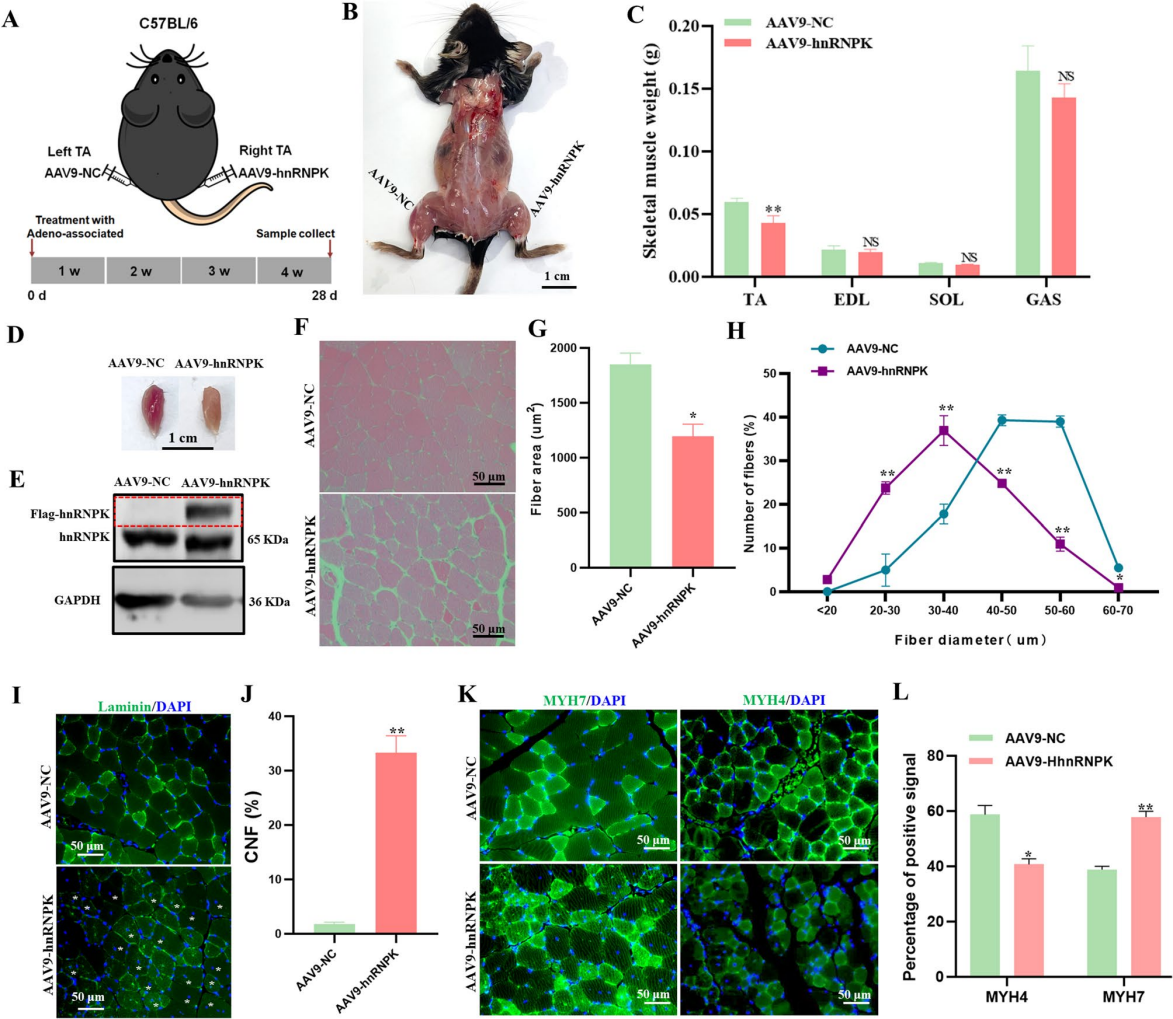
AAV9 mediated overexpression of hnRNPK in skeletal muscle accelerants muscle aging. (**A**) Schematic representation of the experimental design: TA muscles were transduced with an AAV9-NC or an AAV9-hnRNPK. (**B**) Muscle anatomy of mice after 4 weeks of AAV9 injection. The color of TA injected with AAV9-hnRNPK on the right side is slightly lighter than TA injected with AAV9-NC on the left side. (**C**) Muscle mass of TA, EDL, SOL and GAS muscles were comparative between NC and AAV9-hnRNPK group (*n* = 6). (**D**) Comparison of representative samples of dissected TA muscle between NC and AAV9-hnRNPK group. (**E**) Immunoblotting detecting the expression of protein hnRNPK in NC and AAV9-hnRNPK group TA muscle. The red dashed box displays the hnRNPK strip with a flag tag. (**F**) Representative TA muscles stained with hematoxylin and eosin (H&E) from NC and AAV9-hnRNPK group (*n* = 6). (**G**) Quantification of muscle fibre cross-sectional area (SCA) in TA between NC and AAV9-hnRNPK group. (**H**) Frequency of distribution for muscle diameter (µm) of TA muscle between NC and AAV9-hnRNPK group (*n* = 6). (**I**) Representative immunofluoresence staining of Laminin (green) in TA muscle from NC and AAV9-hnRNPK group. (**J**) Quantification of CNF percentage in TA muscles (*n* = 6). (**K**) Immunofluorescence detection of MYH4 (green) and MYH7 (green) expression in TA muscles from WT control and *Hnrnpk* aKO group. (**L**) Rratio quantification of fast and slow muscles in K (*n* = 6)

We hypothesized that insufficient hnRNPK impairs muscle development, as its depletion in skeletal muscle causes atrophy and reduced weight. To test whether increasing hnRNPK expression could enhance muscle development, we packaged a codon-optimized mouse *Hnrnpk* transgene into an AAV9 vector. AAV9-hnRNPK was injected into the right tibialis anterior (TA) muscle of mice, while AAV9-NC (negative control) was injected into the left TA muscle (Fig. 4A and **B**). After 4 weeks, the TA, gastrocnemius (GAS), extensor digitorum longus (EDL), and soleus (SOL) muscles were analyzed. While the weights of the GAS, EDL, and SOL muscles remained unchanged (*p* > 0.05), the TA muscle weight decreased by over 30% in the AAV9-hnRNPK group compared to controls (*p* < 0.05) (Fig. 4C). Additionally, noticeable color differences were observed between the groups (Fig. 4D), suggesting that hnRNPK overexpression does not promote muscle development. Western blot analysis of TA muscle confirmed successful overexpression of hnRNPK, as indicated by a distinct band above the endogenous hnRNPK protein band in the AAV9-hnRNPK group (Fig. 4E). H&E staining revealed that hnRNPK overexpression reduced muscle fiber diameter and caused a 35% decrease in cross-sectional area (CSA) (1851.42 ± 101.41 vs. 1194.93 ± 109.66, *n* = 6) (Fig. 4F and **G**). The AAV9-hnRNPK group exhibited a higher proportion of small-diameter myofibers (< 40 μm) (Fig. 4H) and an increased percentage of central nuclei fibers (CNF) (Fig. 4I and J), indicative of muscle aging. This was further supported by a shift from fast-twitch (type IIB) to slow-twitch (type I) fibers [17]as evidenced by a 20% reduction in MYH4^+^ fibers (*p* < 0.05) and a significant increase in MYH7^+^ fibers (*p* < 0.05) in the AAV9-hnRNPK group compared to controls (Fig. 4K and **L**). These findings align with previous reports that hnRNPK is upregulated in age-related skeletal muscle aging [18, 19]suggesting that excessive hnRNPK expression may be associated with skeletal muscle aging rather than promoting development. This underscores the importance of maintaining normal hnRNPK levels to preserve muscle integrity.

**Fig. 5:**
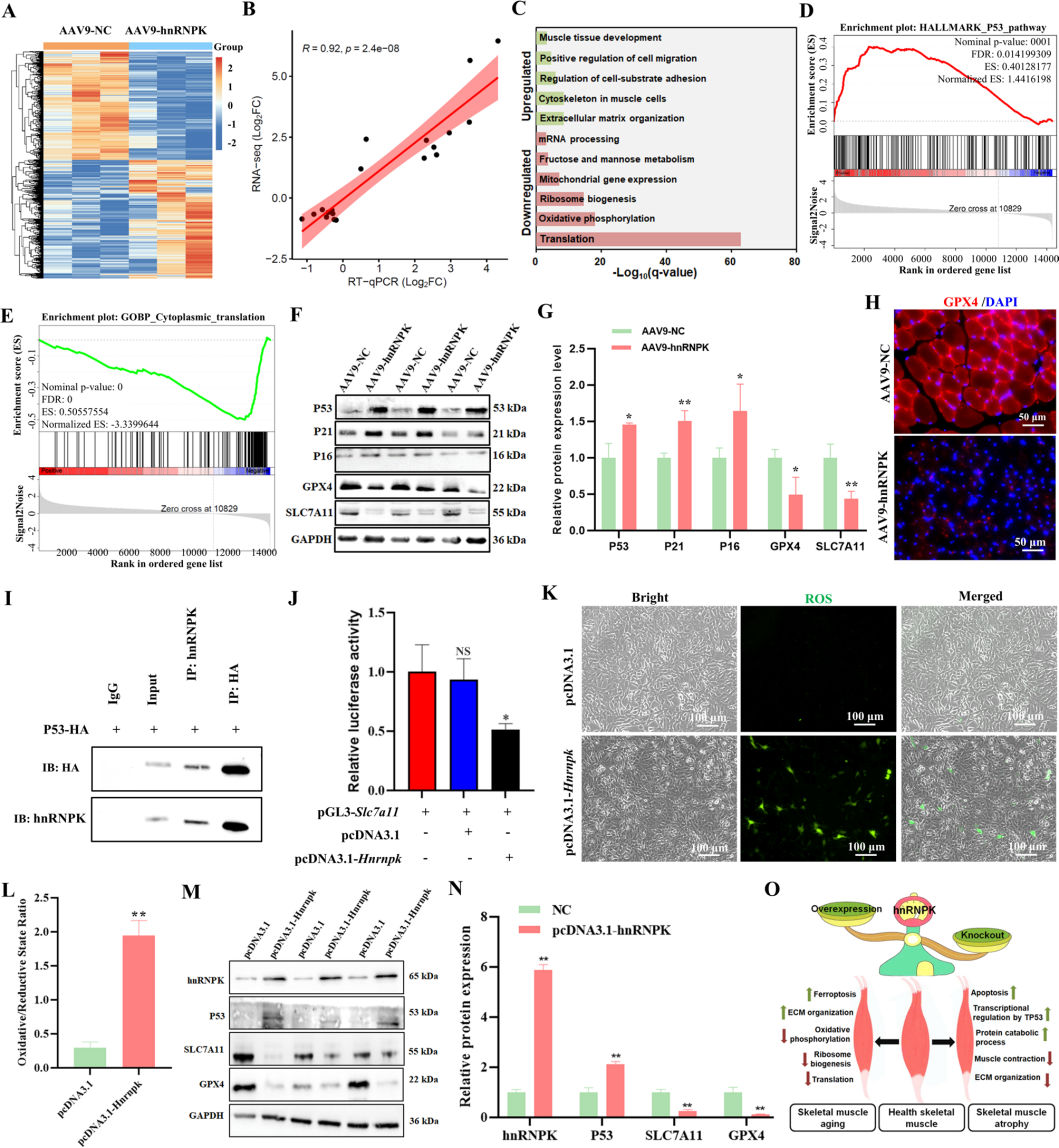
RNA-Seq analysis of the effects of hnRNPK overexpression on skeletal muscle development in mice. (**A**) Heatmap of the expression values of the DEGs in the NC and AAV9-hnRNPK groups TA sample using RNA-Seq. (**B**) Correlation between the fold-change in RNA-Seq analysis and those obtained by RT-qPCR analysis for the 16 selected genes in TA muscles. (**C**) Bar charts showing the enriched GO terms for upregulated and downregulated DEGs between NC and AAV9-hnRNPK groups in TA muscles. **(D**) GSEA of P53 pathway genes in TA muscle upon *Hnrnpk* overexpression. **(E**) GSEA of cytoplasmic translation genes in TA muscle upon *Hnrnpk* overexpression. (**F**) Western blot analysis of P53, P21, P16, GPX4 and SLC7A11 expression in TA muscles from NC and AAV9-hnRNPK groups. **(G**) Statistical analysis of the Western blot results (*n* = 3). All bar and line chart results are expressed as mean values ± SEM, and a paired two-tailed Student’s t-test was used to statistical analysis. ∗∗*p* < 0.01, ∗*p* < 0.05; NS, not significant. (**H**) Immunofluorescence detection of GPX4 (red) expression in TA muscles from NC and AAV9-hnRNPK groups. (**I**) Western blot analysis of immunoprecipitates derived from 293T cells transfected with HA-P53 plasmids. (**J**) *Slc7a11* promoter reporter assays in 293T cell lines following hnRNPK overexpression. 293T cells were transiently transfected with pGL3-*Slc7a11* vector and pcDNA3.1 empty vector or pcDNA3.1-Hnrnpk. Forty eight hours after transfection the cells were harvested and luciferase activity measured and normalized relative to renilla luciferase to control for variations in transfection efficiency. The bars represent the average of three experiments ± SD. ∗*p* < 0.05; ns, not significant. (**K**) Detection of the effect of hnRNPK overexpression on ferroptosis in cells by ROS assay, bar = 100 μm. (**L**) Statistical analysis of the proportion of ROS-positive cells, *n* = 3, **p < 0.01. (**M**) The expression of hnRNPK, P53, SLC7A11 and GPX4 Proteins was analyzed by Western blotting. (**N**) Statistical analysis of relative protein expression gray-value presented in panel **M**. *n* = 3, * *p* < 0.05, ** *p* < 0.01, NS, not significant. (**O**) A schematic illustration showing the phenotypic and transcriptomic signatures of skeletal muscle affected by the hnRNPK expression

To investigate the molecular mechanisms underlying these effects, we performed RNA-Seq on TA tissues from the AAV9-hnRNPK and control groups. This analysis identified 975 upregulated and 1,165 downregulated differentially expressed genes (DEGs) (Fig. 5A and Table S5). Sixteen DEGs were validated by RT-qPCR, showing strong correlation with RNA-Seq data (Pearson *R* = 0.92, *p* = 2.4e-08) (Fig. 5B, Fig. S3C and D). Functional enrichment analysis revealed that upregulated DEGs was primarily associated with extracellular matrix organization, cytoskeletal regulation in muscle cells, cell-substrate adhesion, cell migration, and muscle tissue development. Downregulated DEGs were linked to translation, oxidative phosphorylation, ribosome biogenesis, mitochondrial gene expression, fructose and mannose metabolism, and mRNA processing (Fig. 5C and Table S6). Gene Set Enrichment Analysis (GSEA) further highlighted significant enrichment of altered transcripts in the P53 pathway (Fig. 5D), cytoplasmic translation (Fig. 5E), extracellular matrix binding, oxidative phosphorylation, and ribosome assembly (Fig. S4B). These results suggest that hnRNPK influences muscle development by regulating downstream genes and pathways, particularly through P53-mediated transcription, protein translation, and extracellular matrix assembly.

In light of the established role of hnRNPK overexpression in activating the P53 pathway, which is implicated in muscle aging and ferroptosis [20–22]we investigated its potential involvement in the regulation of ferroptosis. Given that the hnRNPK/P53 pathway is recognized for its inhibitory effect on ferroptosis [20]we postulated that hnRNPK overexpression could influence ferroptosis in skeletal muscle. Our analysis of muscle cells from the AAV9-hnRNPK cohort demonstrated a significant elevation in P53 levels compared to the control group (*p* < 0.05) (Fig. 5F and **G**), aligning with prior research findings. Furthermore, the expression of aging markers P21 and P16 was significantly upregulated in the AAV9-hnRNPK group (*p* < 0.05). In light of the established role of hnRNPK overexpression in activating the P53 pathway, which is implicated in muscle aging and ferroptosis [20–22]we investigated its potential involvement in the regulation of ferroptosis. Given that the hnRNPK/P53 pathway is recognized for its inhibitory effect on ferroptosis [20]we postulated that hnRNPK overexpression could influence ferroptosis pathway in skeletal muscle. Our analysis of muscle cells from the AAV9-hnRNPK cohort demonstrated a significant elevation in P53 levels compared to the control group (*p* < 0.05) (Fig. 5F and **G**), aligning with prior research findings. Furthermore, the expression of aging markers P21 and P16 was significantly upregulated in the AAV9-hnRNPK group (*p* < 0.05). In contrast, the expression of SLC7A11 and GPX4, both of which are negatively regulated by P53 and play critical roles in ferroptosis regulation, were markedly reduced following AAV9-hnRNPK treatment (*p* < 0.05). Immunofluorescence staining corroborated a substantial decrease in GPX4 levels in the skeletal muscle of the AAV9-hnRNPK group relative to controls (Fig. 5H). To further elucidate the transcriptional regulation of *Slc7a11*, we conducted Co-IP assays, which verified the interaction between hnRNPK and P53 (Fig. 5I). A dual-luciferase reporter assay revealed that *Slc7a11* reporter activity was reduced by approximately 50% in hnRNPK-overexpressing 293T cells (Fig. 5J), suggesting that hnRNPK interacts with P53 to negatively regulate *Slc7a11* expression at the transcriptional level. In addition, overexpression of hnRNPK in C2C12 cells significantly increased the proportion of ROS positive cells (*p* < 0.05) (Fig. 5K **and L**). Further Western blot analysis of ferroptosis related protein expression revealed a significant increase in P53 expression (*p* < 0.05), while protein expression of SLC7A11 and GPX4 was significantly reduced (*p* < 0.05) (Fig. 5M **and N**), indicating that overexpression of hnRNPK can induce ferroptosis in C2C12 myocytes through the hnRNPK/Slc7a11/GPX4 axis. Collectively, these findings imply that hnRNPK overexpression in TA muscle may accelerates aging by activating the P53 pathway, enhancing ferroptosis, reducing muscle weight, and impairing ribosome production. Elevated hnRNPK levels disrupt extracellular matrix assembly and protein synthesis, leading to compromised muscle homeostasis and development. However, the precise molecular mechanisms underlying hnRNPK’s role in muscle aging warrant further investigation.

Figure 5O encapsulates our findings, illustrating that both gain- and loss-of-function of hnRNPK result in decreased muscle mass, reduced fiber size, and impaired skeletal muscle homeostasis. These outcomes are mediated by distinct molecular signatures, each linked to specific biological consequences. Notably, hnRNPK deletion is characterized by increased levels of proteins associated with apoptosis, muscle atrophy, and protein catabolic processes, coupled with diminished muscle contraction and ECM organization. Conversely, hnRNPK overexpression is linked to heightened ferroptosis pathway and enhanced ECM organization, alongside reduced oxidative phosphorylation and protein synthesis. Collectively, these findings emphasize the critical importance of precise hnRNPK expression regulation for maintaining skeletal muscle health and preventing pathological conditions related to muscle atrophy. Deviations from optimal hnRNPK levels, whether excessive or deficient, may negatively impact skeletal muscle development.

## Discussion

In this study, we elucidated the indispensable role of hnRNPK in embryonic myogenesis, highlighting its function as a pivotal regulator of skeletal muscle development and homeostasis. By employing a Myf5-Cre-driven conditional knockout mouse model (*Hnrnpk* mKO), we specifically ablated hnRNPK in myoblast precursors, which resulted in pronounced muscle hypoplasia, disrupted fetal myogenesis, and prenatal embryonic lethality. Similarly, using an Acta1-CreEsr1-mediated myofiber-specific inducible knockout model (*Hnrnpk* aKO), we observed that hnRNPK deficiency precipitated global transcriptional dysregulation in skeletal muscle, heightened apoptosis, and severe atrophy. To further delineate the role of hnRNPK, we established an AAV9-mediated skeletal muscle-specific overexpression model. Contrary to our initial hypothesis—that overexpression would yield a phenotype inverse to that of the knockout—both hnRNPK gain- and loss-of-function significantly diminished muscle mass and fiber cross-sectional area, thereby impeding developmental progression. Notably, hnRNPK knockout exerted a more pronounced long-term impact on skeletal muscle development, as evidenced by the mortality observed in mice two weeks following muscle fiber-specific knockout induction. Collectively, these findings underscore the necessity of tightly regulated hnRNPK expression for skeletal muscle development.

Genetic manipulation through knockout and overexpression constitutes a fundamental approach for elucidating gene function. Typically, knockout induces in loss-of-function phenotypes, while overexpression yields gain-of-function effects. Nonetheless, exceptions exist where both types of genetic perturbations yield analogous phenotypic outcomes. For instance, in zebrafish embryos, both knockdown and overexpression of *Unc-45b* similarly disrupt sarcomere organization and reduce myosin expression [23, 24]. In middle-aged mice, both knockdown and overexpression of *Drp1* impair mitochondrial mass, thereby exacerbating skeletal muscle atrophy and autophagic dysfunction [25]. Likewise, in hematopoietic systems, both overexpression and knockout of miR-126 promote leukemogenesis by enhancing the self-renewal of cancer stem cells [26]. These examples underscore the complexity of genetic networks—where dysregulation of gene dosage, rather than simple loss/gain of function, drives phenotypic convergence. Strikingly, the present study reveals a parallel phenomenon: both knockout and overexpression of hnRNPK unexpectedly reduced skeletal muscle weight and fiber size. Mechanistically, hnRNPK knockout promoted atrophy via p53-mediated apoptosis and proteolysis, whereas overexpression triggered ferroptosis through p53-dependent oxidative stress signaling—highlighting p53’s context-dependent role in cell fate decisions. RNA-Seq unveiled reciprocal pathway remodeling: hnRNPK knockout upregulated RNA metabolism and ribosome biogenesis but repressed ECM-receptor interaction pathways, while overexpression activated ECM organization and cytoskeletal signaling at the expense of translational machinery. These findings highlight the multifaceted role of hnRNPK in muscle development, mediated through divergent regulatory axes.

hnRNPK demonstrates context-dependent multifunctionality, functioning as both a tumor suppressor and an oncogene [16, 27, 28]. Clinical and preclinical studies suggest that both the overexpression and underexpression of hnRNPK similarly contribute to the progression of acute myeloid leukemia (AML) [29]. Furthermore, hnRNPK and P53 engage in bidirectional regulation, collaboratively influencing stress-responsive P53 targets while reciprocally modulating each other’s expression [14, 30]. Specifically, hnRNPK can enhance p53 function through transcriptional co-activation, translation regulation, and stability maintenance [31]while p53 can regulate the activity and stability of hnRNPK through transcriptional regulation and SUMOylation modification [32]. In skeletal muscle, both knockout and overexpression of hnRNPK lead to upregulation of p53 expression, highlighting the pleiotropic role of hnRNPK in skeletal muscle biology, which extends beyond simple activation/inhibition paradigms. This complexity underscores the multifaceted role of hnRNPK in muscle development. The bidirectional and multifaceted interaction between hnRNPK and p53 further emphasizes the need to maintain hnRNPK homeostasis for proper muscle development. However, the mechanisms through which hnRNPK dysregulation disrupts myogenesis remain inadequately understood, and the regulatory networks governing its expression during development require further elucidation. Future research should aim to elucidate hnRNPK’s role in skeletal muscle homeostasis using genetically modified murine models and primary satellite cell cultures, combined with functional assays (e.g., ChIP, RNA immunoprecipitation and transcriptomics).

This study provides valuable insights but has limitations. It predominantly concentrates on skeletal muscle, thereby constraining the comprehension of hnRNPK’s functions in cardiac and non-muscle tissues. Although the study identifies hnRNPK’s involvement in the P53, ferroptosis, and ECM pathways is identified, the precise molecular mechanisms remain elusive. To enhance the generalizability of these findings, it is essential to validate them across additional animal models and human tissues. Future research should further investigate the interaction between hnRNPK and P53 in skeletal muscle and other tissues, as well as the regulatory effects of hnRNPK on P53 expression, and elucidate its molecular mechanisms in muscle development and aging, including interactions with downstream genes and pathways. Furthermore, the therapeutic potential of modulating hnRNPK expression warrants exploration. Targeted approaches, such as small-molecule inhibitors or muscle-specific gene therapies, could offer novel treatments for muscle disorders. Finally, further studies should examine the role of hnRNPK in human muscle aging and disease, as this could yield valuable insights for the development of targeted therapeutic interventions.

In summary, this study offers valuable insights into the role of hnRNPK in the development and aging of skeletal muscle. Although the findings hold substantial theoretical and practical significance, additional research is required to comprehensively elucidate the underlying mechanisms and investigate potential therapeutic applications.

## Supplementary Information

Below is the link to the electronic supplementary material.

Supplementary Material 1

Supplementary Material 2

Supplementary Material 3

Supplementary Material 4

Supplementary Material 5

Supplementary Material 6

## Data Availability

No datasets were generated or analysed during the current study.
