# Supplementary material for: The paradox of hnRNPK: both absence and excess impair skeletal muscle function in mice
Source: Skelet Muscle. 2025 Aug 7;15:20. doi: 10.1186/s13395-025-00393-3 (PMC12329970; doi:10.1186/s13395-025-00393-3)
Supplement: Supplementary file 1 — Supplementary Material 1 [file 13395_2025_393_MOESM1_ESM.docx]

**Supplementary figure legends**


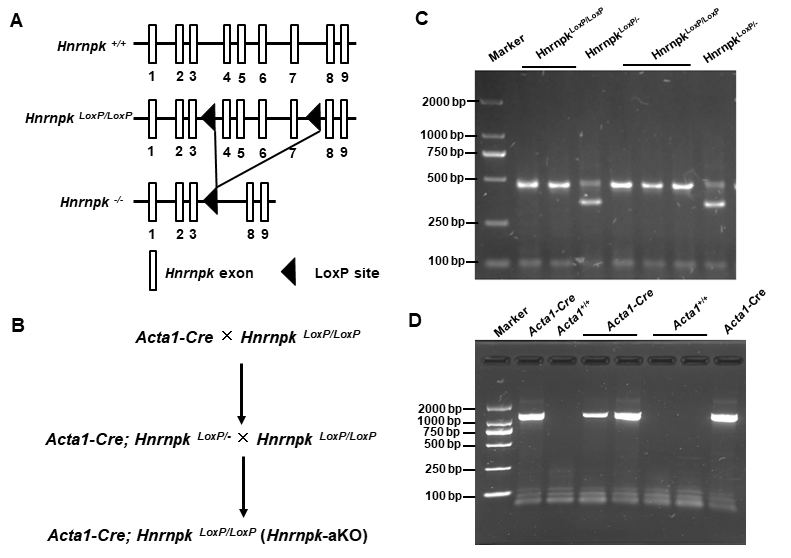


**Fig. S1 Design, genotyping, and knockout of *Hnrnpk* knockout targets in mice.** (**A**) Schematic drawing of the targeting strategy for the generation of *Hnrnpk^LoxP/LoxP^* and *Hnrnpk* muscle fibers-specific knockout (*Hnrnpk* aKO) mice. Exons 4, 5, 6, and 7 were flanked by loxP and will be removed by Cre recombinase. The locations of the primers used for genotyping are shown as bent arrows. (**B**) Mating strategies of *Hnrnpk* aKO mice. (**C**) PCR identification of *Hnrnpk^LoxP/LoxP^* homozygotes. (**D**) PCR identification of *Acta1-CreEsr1* mice.


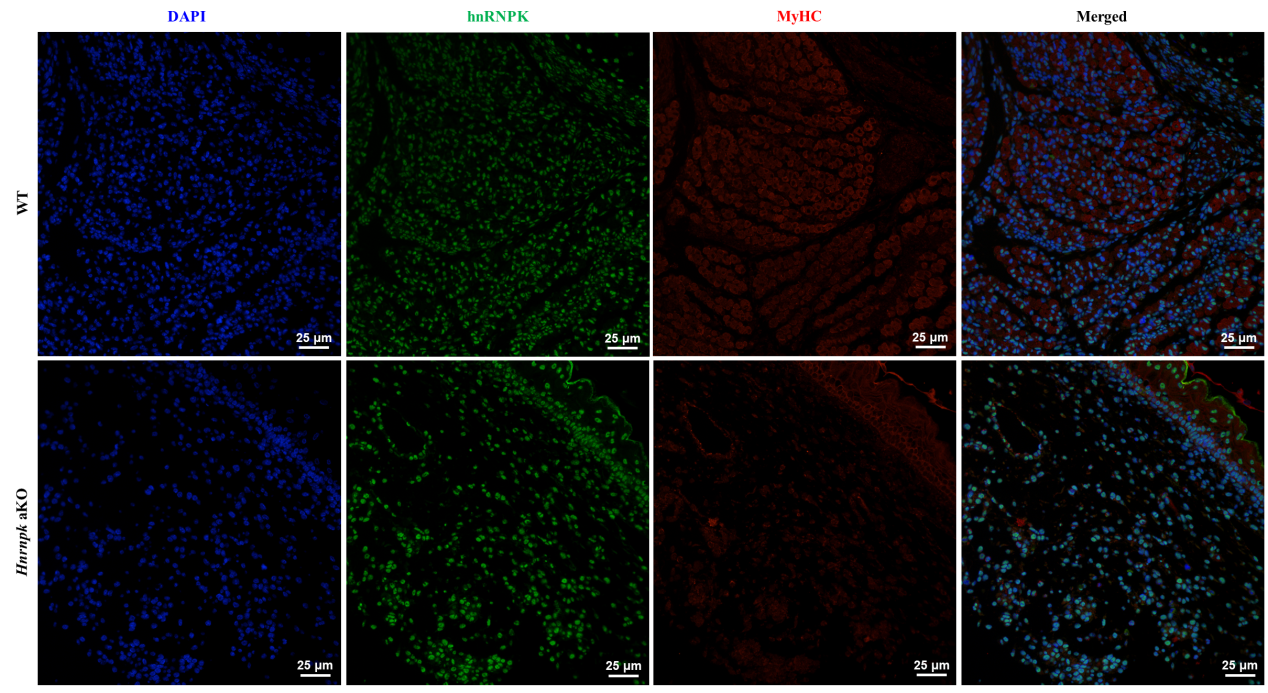


**Fig. S2** Detection of hnRNPK (green) by immunofluorescence (IF) staining in transversal hind limb sections of *Hnrnpk* mKO and control fetuses at E17.5. Muscle fibers were visualized with myosin heavy chain (MyHC) antibody (red), nuclei were stained with DAPI (blue), bar = 25 μm.


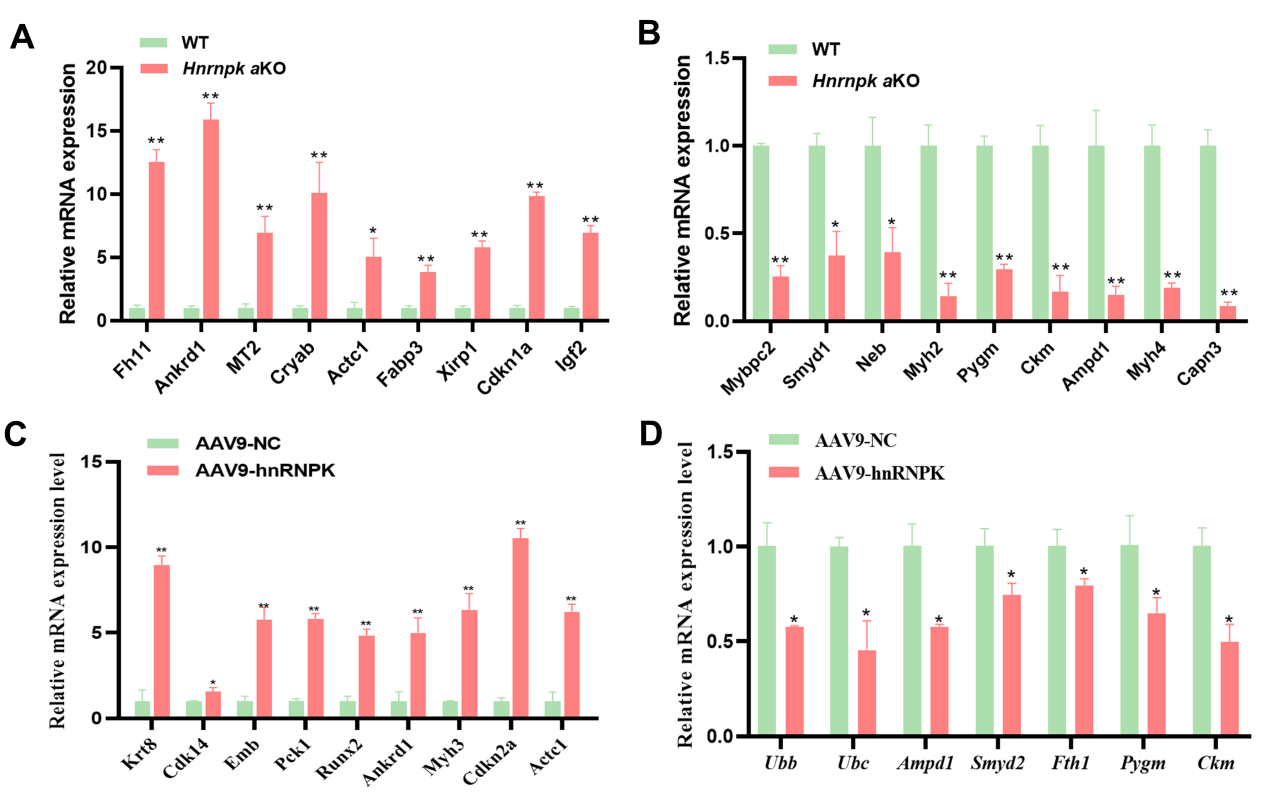


**Fig. S3 Verify RNA-seq results using RT-qPCR.** (**A**) RT-qPCR validation of upregulated DGEs in *Hnrnpk* aKO mice. (**B**) RT-qPCR validation of downregulated DGEs in *Hnrnpk* aKO mice. (**C**) RT-qPCR validation of upregulated DGEs in AAV9-hnRNPK group. (**D**) RT-qPCR validation of downregulated of DGEs in AAV9-hnRNPK group. All results are expressed as mean values ± SEM, and a paired two-tailed Student's t-test was used to statistical analysis. n = 3, ∗∗*p* < 0.01, and ∗*p* < 0.05.

**
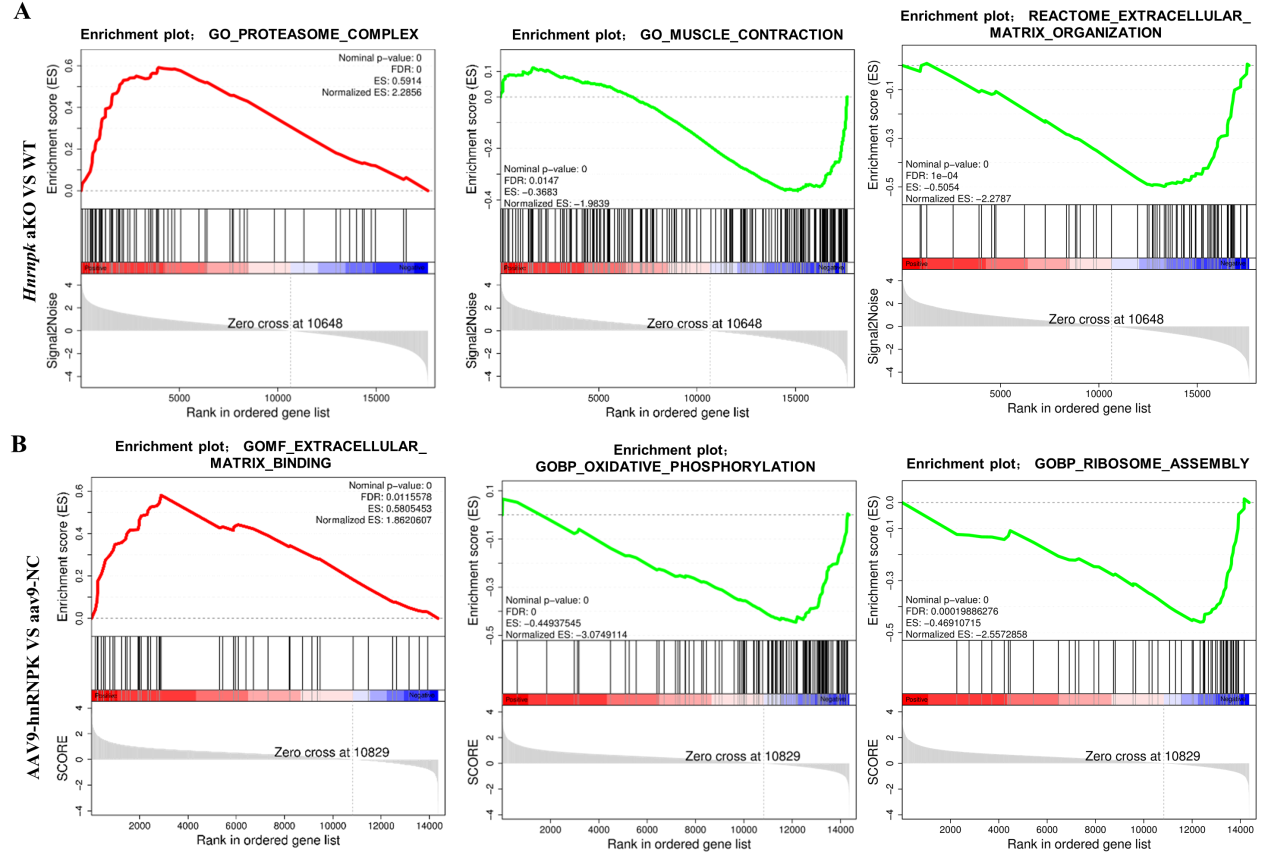
**

**Fig. S4 Main signaling pathways of** **hnRNPK muscle fiber specific knockout or overexpression enrichment analyzed by GSEA. (A**) GSEA analyses of gene sets for interested items in hnRNPK muscle fiber specific knockout, such as proteasome complex, muscle contraction, and extracellular matrix organization. (**B**) GSEA analyses of gene sets for interested items in hnRNPK muscle fiber specific overexpression, such as extracellular matrix binding, oxidative phosphorylation, and ribosome assembly. NES, normalized enrichment score; FDR, false discovery rate. NES, normalized enrichment score; FDR, false discovery rate. Positive and negative NES indicate higher and lower expression in *Hnrnpk* aKO or AAV9-hnRNPK groups, respectively.

**Supplementary table legends**

**Table S1** **Primer sequences for the specific genes of RT-qPCR analysis**

| **Gene** | **Primer sequence（5’-3’）** | **Tm (℃)** | **Length（bp）** | **GenBank Accession No** |
| --- | --- | --- | --- | --- |
| *Hnrnpk loxP* | F: GTCTCTCGCTCTGTCTTTGTGGC  R: GGAAGGGCTCAGATTAAGTGGCAA | 58 | 473 or 360 | NC_000079 |
| *Myf5-cre* | F: AACCAGAGACTCCCCAAGGT  R1: CGGCTCTTAAAGCAATGGTC  R2: ACGAAGTTATTAGGTCCCTCGAC | 60 | 120 or 240 |  |
| *ACTA1-CreEsr1* | F: CGAGCCGAGAGTAGCAGTTGTA  R: AGGTGGACCTGATCATGGAG | 62 | 1100 |  |
| *Fhl1* | F: TGCTTTGACAAGTTCTGCG  R: CTTATAATGCACCTCCTTGGC | 60 | 81 | [NM_001412081.1](https://www.ncbi.nlm.nih.gov/entrez/viewer.fcgi?db=nucleotide&id=2302515520) |
| *Ankrd1* | F: CTGCGATGAGTATAAACGGAC  R: GCCTCCATTAACTTCTCCAC | 60 | 81 | [NM_013468.3](https://www.ncbi.nlm.nih.gov/entrez/viewer.fcgi?db=nucleotide&id=133893293) |
| *Mt2* | F: TGCAAATGTACTTCCTGCA  R: GAAGCCTCTTTGCAGATGC | 60 | 92 | [NM_008630.2](https://www.ncbi.nlm.nih.gov/entrez/viewer.fcgi?db=nucleotide&id=123701854) |
| *Cryab* | F: TCTCAGAGATGCGTTTGGA  R: TGACTTTGAGTTCCTCCGG | 60 | 81 | [XM_036154592.1](https://www.ncbi.nlm.nih.gov/entrez/viewer.fcgi?db=nucleotide&id=1907194824) |
| *Actc1* | F: GCCCTCTTTCATTGGTATGG  R: GCGGATATCGATGTCACAC | 60 | 82 | [NM_009608.4](https://www.ncbi.nlm.nih.gov/entrez/viewer.fcgi?db=nucleotide&id=918409972) |
| *Fabp3* | F: CATGTGCAGAAGTGGAACG  R: ATGAGTGAGAGTCAGGATGAG | 60 | 81 | [NM_010174.2](https://www.ncbi.nlm.nih.gov/entrez/viewer.fcgi?db=nucleotide&id=1686254194) |
| *Xirp1* | F: CCAGACCCTTGAAGGATGG  R: CAGGTCCTCTTCTGTCCTC | 60 | 81 | [NM_011724.5](https://www.ncbi.nlm.nih.gov/entrez/viewer.fcgi?db=nucleotide&id=2238885702) |
| *Cdkn1a* | F: TACCGTGGGTGTCAAAGCA  R: AGGGAGGGAGCCACAATAC | 60 | 128 | [NM_001111099.2](https://www.ncbi.nlm.nih.gov/entrez/viewer.fcgi?db=nucleotide&id=930425325) |
| *Pygm* | F: TGAGAAAGTTATTCCCGCC  R: GAACTTCATGTTGCCAGTG | 60 | 85 | [NM_011224.2](https://www.ncbi.nlm.nih.gov/entrez/viewer.fcgi?db=nucleotide&id=957801613) |
| *Mybpc2* | F: GCAGGTATTCGTAGGTGAC  R: CCATCTTTCATCCACATAACCT | 60 | 81 | [NM_146189.3](https://www.ncbi.nlm.nih.gov/entrez/viewer.fcgi?db=nucleotide&id=289666785) |
| *Ckm* | F: GAACCTCAAGGGTGGAGAC  R: GTGTAACCCTTGATGCTGC | 60 | 84 | [NM_007710.2](https://www.ncbi.nlm.nih.gov/entrez/viewer.fcgi?db=nucleotide&id=118131179) |
| *Myh2* | F: TCAGGCTTCAGGATTTGGT  R: ATTGTTCCTCAGCCTCCTC | 60 | 81 | [NM_001039545.2](https://www.ncbi.nlm.nih.gov/entrez/viewer.fcgi?db=nucleotide&id=205830427) |
| *Myh4* | F: GTCAGTCAAGGTGAAATCACAG  R: CAGGATATCAACAGCAGTGTC | 60 | 81 | [NM_010855.3](https://www.ncbi.nlm.nih.gov/entrez/viewer.fcgi?db=nucleotide&id=444299629) |
| *Neb* | F: TACCATTACAGGTCTGCGT  R: GATTGAATGTTGTCTTCTGCC | 60 | 81 | [XM_006497776.2](https://www.ncbi.nlm.nih.gov/entrez/viewer.fcgi?db=nucleotide&id=1907136520) |
| *Smyd1* | F: ATCACGAGGTTGTGAAGCT  R: GAAGCACGTAGAGGTTGGT | 60 | 81 | [NM_009762.2](https://www.ncbi.nlm.nih.gov/entrez/viewer.fcgi?db=nucleotide&id=236461141) |
| *Capn3* | F: TGTCCTCAACACAGTGGTG  R: AGCCATCTGTATCCATGAGAG | 60 | 101 | [NM_001109761.2](https://www.ncbi.nlm.nih.gov/entrez/viewer.fcgi?db=nucleotide&id=295317370) |
| *Ampd1* | F: CTGAGAAAGGAACGAGGCA  R: TGTCATGAGGTGAGTGAGAG | 60 | 81 | [XM_006501400.5](https://www.ncbi.nlm.nih.gov/entrez/viewer.fcgi?db=nucleotide&id=1907150344) |
| *Emb* | F: ATCGCTTACGTGGGGGATTC  R: GAGCGTCAATGGGAACCTGT | 60 | 112 | [NM_010330.4](https://www.ncbi.nlm.nih.gov/entrez/viewer.fcgi?db=nucleotide&id=226437628) |
| *Myh3* | F: TGTCACAGTCAGAGGTCCCA  R: AAGGGCTGGTTCTGAGCTTC | 60 | 172 | [XM_006532412.2](https://www.ncbi.nlm.nih.gov/entrez/viewer.fcgi?db=nucleotide&id=1907080344) |
| *Krt8* | F: TCCTTCATTGACAAGGTGCG  R: ATGTTGCTCCTCGACGTCTT | 60 | 98 | [NM_031170.2](https://www.ncbi.nlm.nih.gov/entrez/viewer.fcgi?db=nucleotide&id=114145560) |
| *Ryr1* | F: ACACTCAAGACTCTGCTGG  R: CTTCGTCTTCTTCAGGTTGTC | 60 | 81 | [XM_036152819.1](https://www.ncbi.nlm.nih.gov/entrez/viewer.fcgi?db=nucleotide&id=1907178626) |
| *Pck1* | F: GGGTGGAAGGTCGAATGTGT  R: AGCCCTTAAGTTGCCTTGGG | 60 | 72 | [NM_011044.3](https://www.ncbi.nlm.nih.gov/entrez/viewer.fcgi?db=nucleotide&id=1380941588) |
| *Cdk14* | F: CCCAGATGTGCGACCTCATT  R: CTTTCTTCAGAGCGATGCGG | 60 | 108 | [NM_011074.4](https://www.ncbi.nlm.nih.gov/entrez/viewer.fcgi?db=nucleotide&id=2581710095) |
| *Cdkn2a* | F: TGGTCACTGTGAGGATTCAGC  R: TTGCCCATCATCATCACCTGG | 60 | 191 | NM_009877.2 |
| *Runx2* | F: AGTGCGGTGCAAACTTTCTC  R: CTTGCAGCCTTAAATGACTCGG | 60 | 115 | NM_009820.6 |
| *Ubb* | F: CGAGAGGCTTTGTCCGGTT  R: GTGATGAAAGCACAAACCGCC | 60 | 75 | NM_011664.5 |
| *Ubc* | F: CCACCAAGAAGGTCAAACAGGA  R: ACACCCAAGAACAAGCACAAG | 60 | 87 | NM_019639.4 |
| *Smyd2* | F: TTGGGACTCCAGCCTTCCT  R: CTTCAAGGAACTCGGCGCT | 60 | 153 | XM_006497146.5 |
| Fth1 | F: TTTGCAACTTCGTCGTTCCG  R: CAGGTTGATCTGGCGGTTGA | 60 | 141 | [NM_010239.2](https://www.ncbi.nlm.nih.gov/entrez/viewer.fcgi?db=nucleotide&id=407027852) |
| *Murf1* | F: GAGGGCCATTGACTTTGGGA  R: TCCAGAGCGTGTCTCACTCA | 60 | 141 | [NM_001039048.2](https://www.ncbi.nlm.nih.gov/entrez/viewer.fcgi?db=nucleotide&id=124244069) |
| *Msfbx* | F: CGGGGTTTGTTTTCAGCAGG  R: ACACAGACATTGCCTCCCAG | 60 | 153 | [NM_026346.3](https://www.ncbi.nlm.nih.gov/entrez/viewer.fcgi?db=nucleotide&id=341926178) |
| *Bax* | F: GCACGTCCACGATCAGTCAC  R: ACCCTGTAGCAAAAAGGCCC | 60 | 163 | [XM_011250780.4](https://www.ncbi.nlm.nih.gov/entrez/viewer.fcgi?db=nucleotide&id=1907176875) |
| *Casp3* | F: CATTGACGATGGCACGAACA  R: GACGGTCTGTCATCTGAGCC | 60 | 147 | [NM_007601.3](https://www.ncbi.nlm.nih.gov/entrez/viewer.fcgi?db=nucleotide&id=295317369) |
| *Capn3* | F: AGCTTGGAACGGTACGCTAA  R: GAGTCCACTGACTTGCTCCC | 60 | 117 | [NM_001284409.1](https://www.ncbi.nlm.nih.gov/entrez/viewer.fcgi?db=nucleotide&id=549806821) |
| *18S rRNA* | F: ACCGCAGCTAGGAATAATGGA  R: GCCTCAGTTCCGAAAACCA | 60 | 63 | NR_003278.3 |
| *Gapdh* | F: AGGTCGGTGTGAACGGATTTG  R: GGGGTCGTTGATGGCAACA | 60 | 95 | NM_001289726.1 |

**Table S2 Primary antibody list**

| **Antibody** | **Resource** | **Usage and dilution** |
| --- | --- | --- |
| Anti-hnRNPK | Abcam, ab39975 | IF, 1:200; IP, 1:100 |
|  | Beyotime, AF1972 | WB, 1:1000 |
| Anti-GPX4 | Servicebio, GB114327 | IF, 1:1000 |
|  | Beyotime, AF7020 | WB, 1:1000 |
| Anti-MURF1 | Proteintech, 55456 | IF, 1:50  WB, 1:15000 |
| MAFbx | Proteintech, 67172 | IF, 1:200  WB, 1:5000 |
| Anti-P53 | Proteintech, 60283 | WB, 1:500 |
| Anti-P21 | Beyotime, AP021 | WB, 1:200 |
| Anti-P16 | Beyotime, SF6471 | WB, 1:800 |
| Anti-SLC7A11 | Proteintech, 26864 | WB, 1:800 |
| Anti-MYH7 | Beyotime, AF7533 | IF, 1:50 |
| Anti-MYH4 | Santa, sc-32732 | IF, 1:100 |
| Anti-Laminin | Sigma, L9393 | IF, 1:200 |
| Anti-γH2A.X | Beyotime, AG2114 | WB, 1:2000 |
| Anti-GAPDH | Proteintech, AF0006 | WB, 1:10000 |
| Anti-HA | Beyotime, AG8057 | IP, 1:100 |
