# Supplementary figures and images for: The paradox of hnRNPK: both absence and excess impair skeletal muscle function in mice
Source: Skelet Muscle. 2025 Aug 7;15:20. doi: 10.1186/s13395-025-00393-3 (PMC12329970; doi:10.1186/s13395-025-00393-3)

**Fig. 2F**

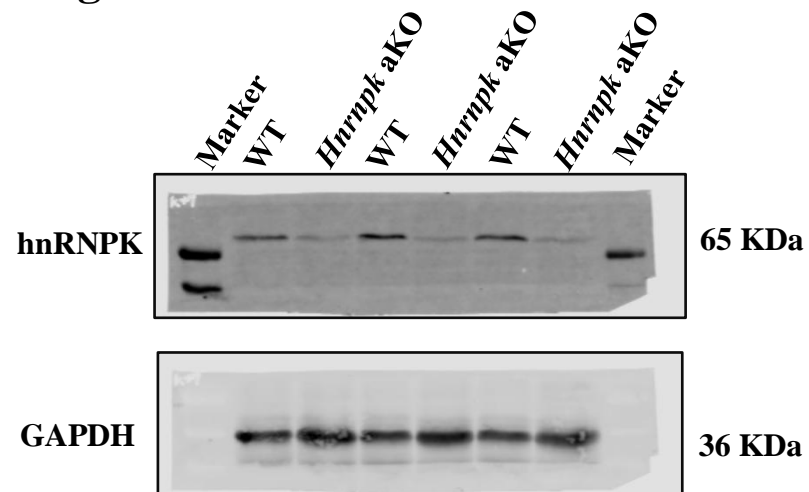

**Fig. 3G**

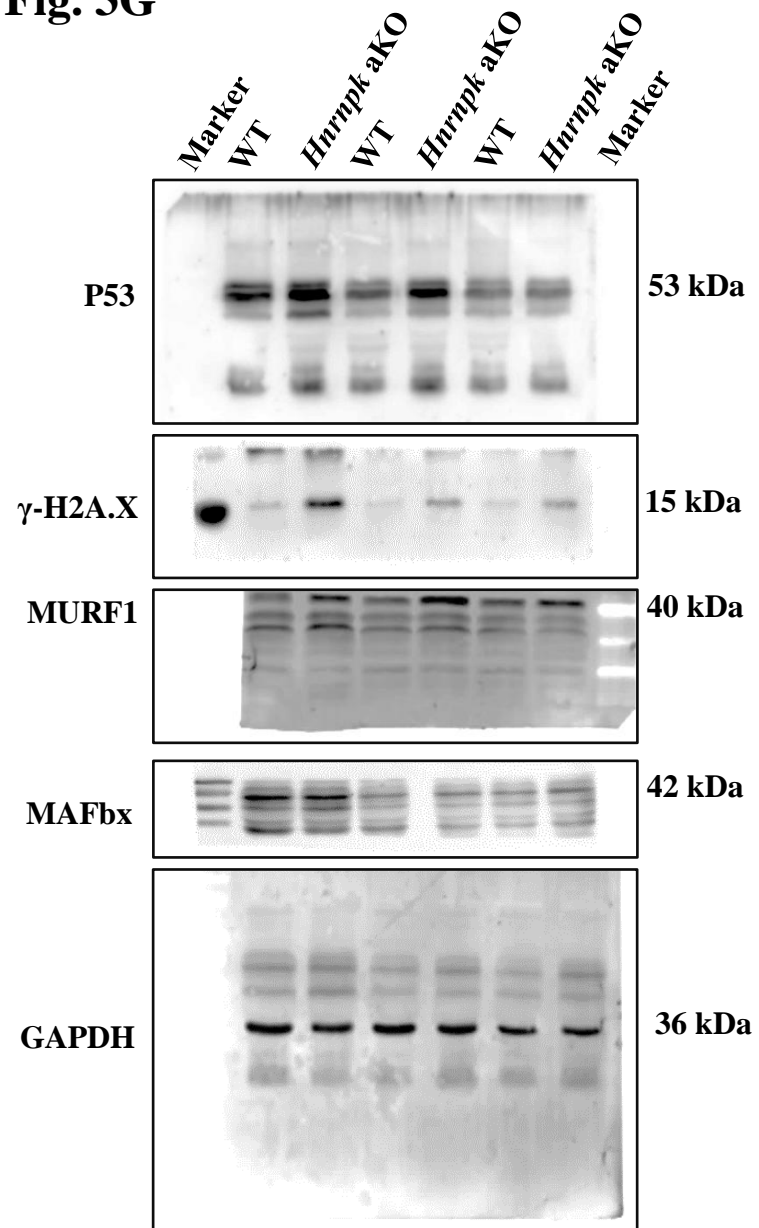

**Fig. 4E**

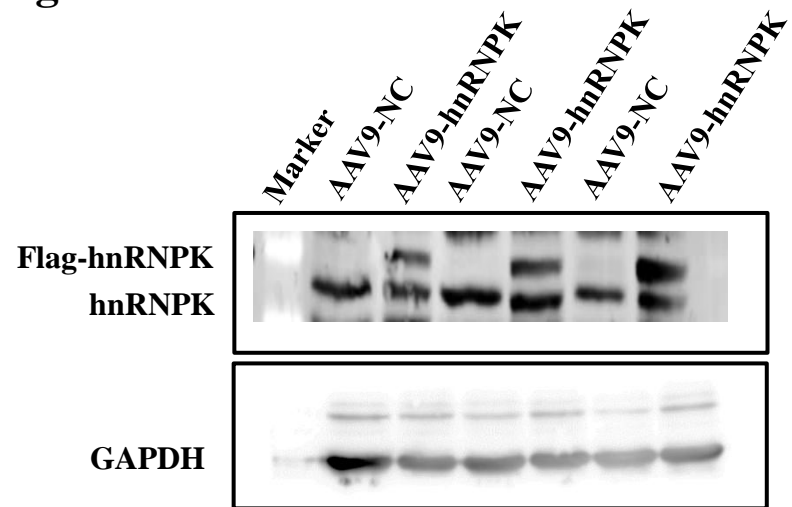

**Fig. 5F**

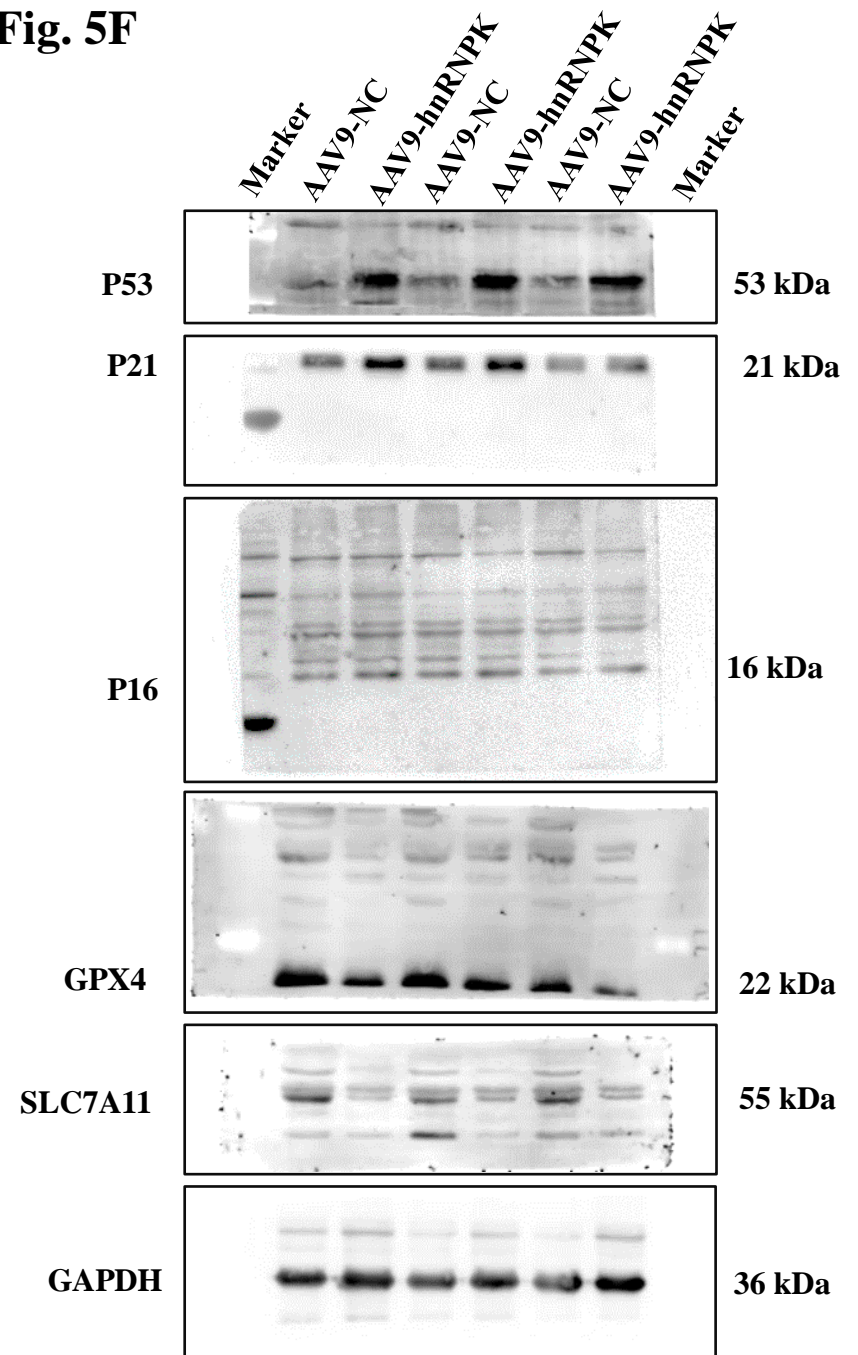

**Fig. 5I**

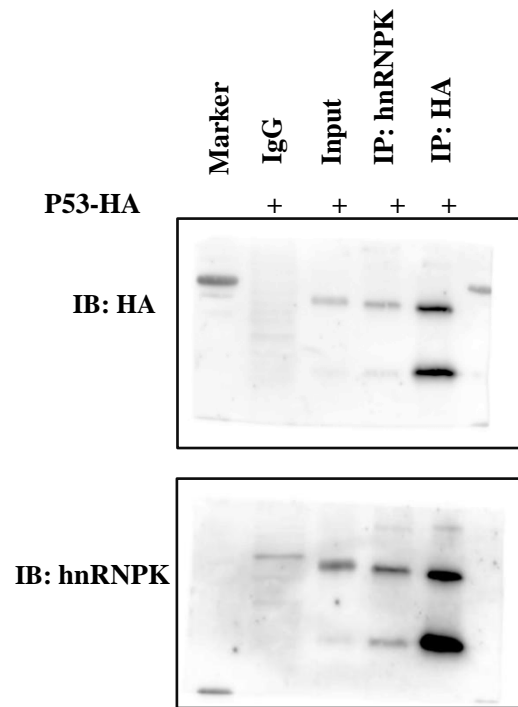

**Fig. 5M**

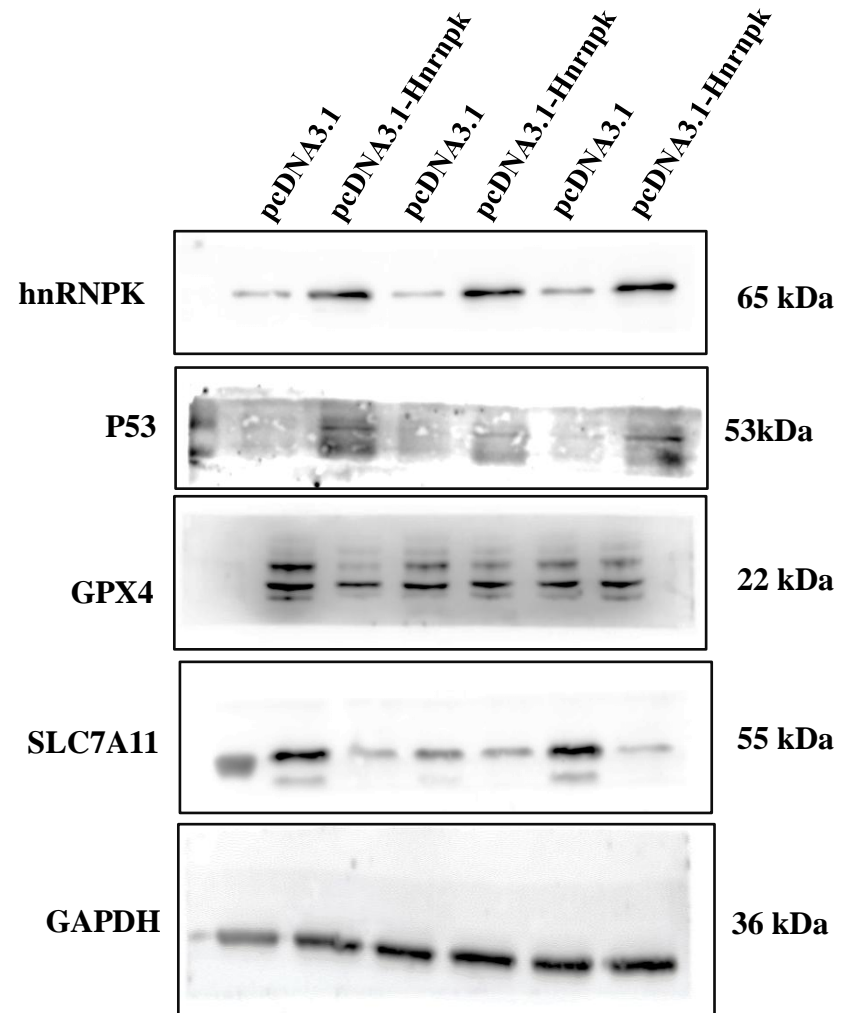

Supplement: Supplementary file 6 — Supplementary Material 6 [file 13395_2025_393_MOESM6_ESM.pdf]
